# Persistence of parental age effect on somatic mutation rates across generations in *Arabidopsis*

**DOI:** 10.1186/s12870-023-04150-w

**Published:** 2023-03-22

**Authors:** Shashi Bhushan, Amit Kumar Singh, Yogendra Thakur, Ramamurthy Baskar

**Affiliations:** 1 Department of Biotechnology, Bhupat and Jyoti Mehta School of Biosciences, Indian Institute of Technology-Madras, Chennai, 600 036 India; 2 Department of Plant Molecular Biology, University of Delhi South Campus, Benito Juarez Road, New Delhi, 110021 India; 3 Institut de Biologie Moléculaire des Plantes, UPR2357 CNRS, Université de Strasbourg, 12 rue du Général Zimmer, Strasbourg Cédex, 67084 France

**Keywords:** Arabidopsis, DNA repair, Epigenetic, GUS expression, Parental age, Methylation, Somatic mutation

## Abstract

In the model plant *Arabidopsis thaliana*, parental age is known to affect somatic mutation rates in their immediate progeny and here we show that this age dependent effect persists across successive generations. Using a set of detector lines carrying the mutated *uidA* gene, we examined if a particular parental age maintained across five consecutive generations affected the rates of base substitution (BSR), intrachromosomal recombination (ICR), frameshift mutation (FS), and transposition. The frequency of functional GUS reversions were assessed in seedlings as a function of identical/different parental ages across generations. In the context of a fixed parental age, BSR/ICR rates were unaffected in the first three generations, then dropped significantly in the 4th and increased in most instances in the 5th generation (e.g. BSR (F1 38 = 0.9, F2 38 = 1.14, F3 38 = 1.02, F4 38 = 0.5, F5 38 = 0.76)). On the other hand, with advancing parental ages, BSR/ICR rates remained high in the first two/three generations, with a striking resemblance in the pattern of mutation rates (BSR (F1 38 = 0.9, F1 43 = 0.53, F1 48 = 0.79, F1 53 = 0.83 and F2 38 = 1.14, F2 43 = 0.57, F2 48 = 0.64, F2 53 = 0.94). We adopted a novel approach of identifying and tagging flowers pollinated on a particular day, thereby avoiding biases due to potential emasculation induced stress responses. Our results suggest a time component in counting the number of generations a plant has passed through self-fertilization at a particular age in determining the somatic mutation rates.

**Supplementary Information:**

The online version contains supplementary material available at 10.1186/s12870-023-04150-w.

## Introduction

Adaption during evolution heavily relies on variations within the DNA sequence [1] and somatic mutations are an important source of genetic variation in plants [2, 3]. A fraction of such spontaneous mutations are known to be transmitted from reproductive tissues arising later in development [4] and thus, it is important to understand the frequency and regulators of such events. Spontaneous mutations largely arise as a consequence of impaired proof-reading activity and errors occurring during DNA repair [5, 6]. In *A. thaliana*, the single nucleotide mutation (SNM) rate is around 6.95 × 10^− 9^ per site per generation, occurring mostly within transposable elements (TEs) residing in centromeric regions [7]. Plants are constantly exposed to biotic and abiotic stresses, triggering different classes of somatic mutations [8]. Stress of different kind experienced by organisms are known to affect the phenotypic traits of the offspring. Thus the phenotype of an organism is also determined by the impact of various environmental/growth conditions experienced in earlier generations [9–11]. Such experiences are recorded in the somatic cell lineage, affecting the genome stability to confer better adaptation to the same stress in subsequent generations [4, 8, 12, 13]. Thus, any reproductive cell derived from such somatic tissues would also transfer this recorded memory to the subsequent generation [8].

The induced somatic mutation rates in *Arabidopsis* are reported to persist for up to 4th generations [8], but other studies contradict the existence of this phenomenon [14, 15]. Furthermore, there are also reports suggesting that such transgenerational effects depend on the nature of the stress [14]. Although transgenerational effects are known to be inherited through both the parents in a dominant manner [8] some studies indicate that the influence of maternal gametes on the transgenerational effect is more significant than paternal gametes [13, 16]. Transgenerational effects are context-dependent and their reproducibility is reported to be low [15].

Previously, we reported that parental age of *Arabidopsis* influenced somatic mutation rates in their immediate progeny [17] and here demonstrate the persistence of this effect in its transgenerational progenies as well. Parental age at the time of reproduction has a strong influence on the patterns of somatic mutation across generations and this age-dependent information persists for a few generations after which there seems to be resetting of mutation rates.

## Results

**Fig. 1:**
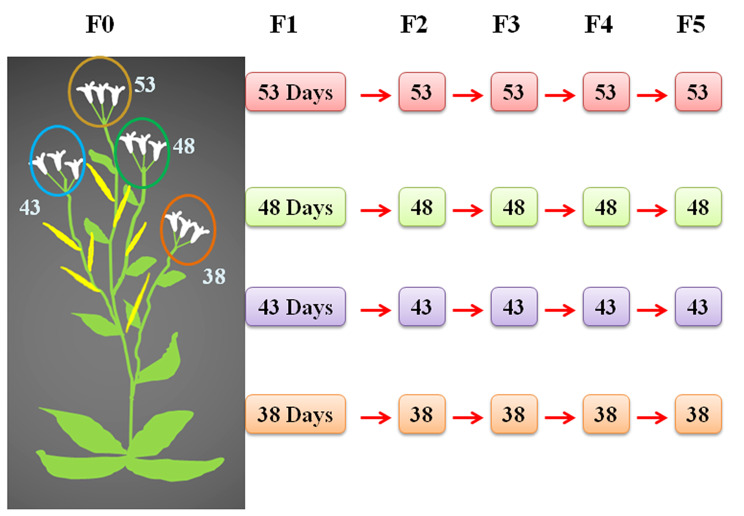
Selfing scheme: Flowers of different DAS were marked with different colored threads and in the subsequent generations also, the self-pollinating age was retained

Using a set of *Arabidopsis* mutation detector lines [18–21] we examined if a particular parental age affected the spontaneous base substitution rates (BSR), intrachromosomal recombination (ICR), frameshift mutation (FS) and transposition rates in the next five consecutive generations. Although the reporter lines are useful in scoring mutation events under different circumstances, they do not necessarily represent the mutation frequency in the entire genome. The reversion rates vary depending on where the mutation has been engineered within the *uidA* and the position of the transgene in the genome [22]. Self-pollinated plants of four different ages (38, 43, 48, and 53 days after sowing (DAS)) were grown, following which the respective reproductive ages were retained in the subsequent generations also (Fig. 1). For every generation and age, we began with 24 plants all obtained from 24 separate plants from the earlier generation. For example, we tagged 20–25 flowers from 24 plants representing 38 DAS and in the subsequent four generations also, we tagged the same number of flowers in the same age. This protocol was followed for other ages in all the generations. The mutation rates were examined in two different ways. (1) Counting reversion events in seedlings obtained from plants of the same age set as depicted in Fig. 1. For instance, mutation rates were compared in seedlings of 38 DAS plants in five consecutive generations (comparison of the same age (same colors) shown in Fig. 1). (2) Furthermore, we compared the mutation rates as a function of advancing parental age across generations. For e.g., the mutation rates were compared across different ages (colors) of the same generation, i.e. 38, 43, 48, and 53 DAS plants among F1 as in Fig. 1.

### Normalization of mutation rates after correcting the cell number and ploidy levels

Mutation rates were calculated based on the reversion of a mutated or truncated *uidA* gene to wild type sequence whereby the acquisition of functional GUS activity results in the development of blue colored spots upon histochemical staining. Thus, the overall reversion frequency was estimated via quantification of the number of such blue spots with respect to the total number of seedlings screened. Since ploidy levels, cell size, and their number vary significantly from seedlings obtained from plants of different ages [17] normalization of the genome per nucleus is required. Plant cells are known to be uneven in size, even within a particular tissue [23]. In the leaf epidermal cells of *Arabidopsis*, a strong correlation exists between the size of the cell and the amount of DNA [24]. Plant cells frequently undergo endoreduplication, resulting in higher ploidy per nucleus which is correlated with increased cell size as well [23, 25]. As a result, the individual cell size and their numbers significantly contribute to the overall leaf size [26]. As the ploidy per nucleus, the cell size, and cell number can vary in seedlings derived from parents of different ages/generations, it is essential to normalize the number of genomes per nucleus. As the leaf size is largely determined by the epidermis [27, 28] the fourth true leaf (excluding the cotyledons) of Columbia (Col-0) wild type plants were used to determine cell size and number [17]. We used a scanning electron microscope (SEM) to count the number of adaxial epidermal cells in a specified area of the leaf and measured the cell size. Thus, the mutation rates were normalized per haploid genome as estimated from cell number and average ploidy in the 4th true leaf.

As a function of constant or advancing parental ages, there was wide variation in the epidermal cell number (Fig. S1, E, and F), and such differences were not apparent in the context of cell size (Fig. S1, G, and H). Interestingly, as a function of fixed parental age, the epidermal cell number increases in the 4th and drops significantly in the 5th generation (Fig. S1, E), although this largely depends on the age group. Consistently across all parental ages, the fraction of 2X nuclei increased in the fourth generation alone (Fig. S2, A) and the percentage of nuclei with different ploidy levels remained more or less constant in the first three age sets (Fig. S2, B) regardless of the generation. Depending on age/generation, there were differences in ploidy levels per leaf cell nucleus, and in older ages (53 DAS) (Fig. S2, C, and D), the variations in ploidy levels were higher compared to subsequent generations (Fig. S2, C). Hence, the mutation rates were corrected for differences in genome numbers (obtained from average ploidy per nucleus) and cell numbers (Table S1).

### Comparsion of reversion rates after manual and natural selfing

Throughout the study, self pollinated flowers of particular DAS were tagged with colored threads to avoid a potential emasculation indued stress response which may distort the mutation rates (Fig. S3). Accordingly, the mutation rates were compared between two sets of seedlings one derived from individual plants manually self-pollinated on different DAS, while the other was obtained from a single plant, but from flowers of different DAS marked with colored threads. Although the pattern of mutation rates after normalization were largely similar, a 0.2 to 0.5 fold higher BSR, ICR, and FS mutation rates were observed in seedlings from manually pollinated plants of advanced ages (Fig. S3) and this increased reversion rates may possibly be due to the result of repeated physical contacts followed by emasculation triggering a stress response.

### As a function of constant parental age, C to T transitions (BSR) are stable in first three consecutive generations

**Fig. 2:**
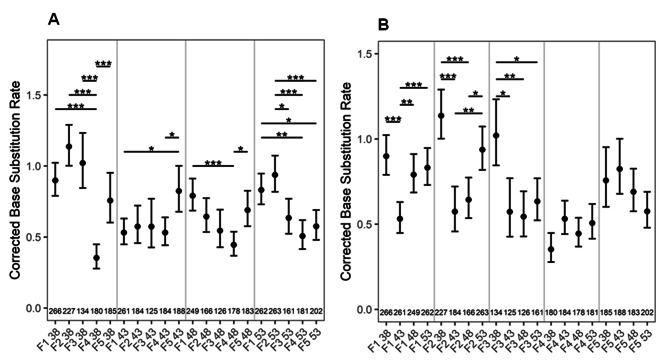
A-B: BSR as a function of (**A**) constant, (**B**) different parental age across different generations. The numbers along the X axis shows the number of seedlings analyzed. *P* values were corrected for multiple testing. No asterisk denotes no significant difference *. *P <* 0.05; **, *P* < 0.01; ***, *P* < 0.001

To determine the frequency of C to T transitions as a function of constant or different parental ages across generations, we used the 1390_T − C_ detector line. In the open reading frame of the *uidA* gene, T is mutated to C at the 1390th position [21]. The *uidA* gene activity can only be regained by a precise reversion of mutation (C to T). In seedlings of 38, 43, and 48 DAS plants, the variation in BSR rates were not significant in the first three generations. However, in the 4th generation, seedlings from 38 to 48 DAS plants exhibited a significant drop in BSR, and thereafter, a significant increase in the 5th generation (Fig. 2A). Similarly, in seedlings from 43 DAS plants, a surge in BSR was observed in the 5th generation (Fig. 2A). Except for the seedlings obtained from the 53 DAS plants, the BSR increased in the 5th generation consistently. Taken together, our results suggest that BSR significantly depends on the number of generations a plant has undergone selfing at a particular age.

### Younger parents produce offsprings with more BSR

By comparing mutation rates across different ages in five consecutive generations, we observed high BSR in the first three generations of seedlings from 38 DAS plants compared to other ages (Fig. 2B). The BSR dropped significantly in seedlings of 43 DAS plants, and increased thereafter as a function of advancing parental ages (48 and 53 DAS). Surprisingly, this pattern was observed only in the first two generations. In the 4th and 5th generations, there was no significant change in BSR, irrespective of parental age (Fig. 2B). These results reiterate the notion that BSR is strongly impacted by the the number of generations a plant has gone through selfing at a particular age.

### ICR rates are stable as a function of constant parental age in three consecutive generations

**Fig. 3:**
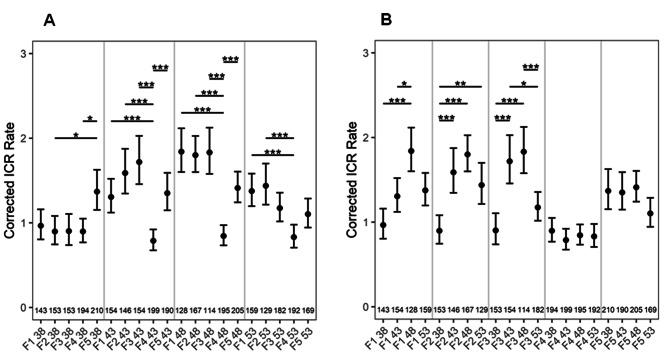
**A-B**: ICR rates as a function of (**A**) constant, (**B**) increasing parental age across different generations. The numbers at the bottom of the graph show the number of seedlings analyzed. *P* values were corrected for multiple testing. No asterisk represents no significant difference. *, *P <* 0.05; **, *P* < 0.01; ***, *P* < 0.001

To examine the effect of reproductive age on ICR rates across generations, we utilized the detector line R2L1, containing two inverted catalase introns within the gene *uidA*. In this system, a recombination event between similar sequences of the catalase intron would result in a functional *uidA* gene, leading to the GUS expression [19]. With a constant age of 38 DAS, there was no significant change in ICR in the first four generations, but the rates significantly increased in the 5th generation (Fig. 3A). However, when the parental age was retained at 43 or 48 DAS, there was no change in ICR rates in the first three generations, following which it decreased in 4th but again surged in the 5th generation. Although such a pattern was also observed in seedlings obtained from 53 DAS plants, the increase in ICR rates was not significant in the 5th generation.

Strikingly, like BSR, ICR rates shoot up in the 5th generation following the aforementioned pattern, and this phenomenon was observed across all ages. This suggests that ICR rates are determined by the number of generations a plant has been previously selfed at a particular reproductive age (Fig. 3A). Interestingly, there is a sigmoidal distribution of mutation rates upon comparison between different age sets across all generations (Fig. 3A).

### ICR rates increase with increasing parental age, but drops in very old ages

Although, the increase in ICR rates (from seedlings of 38, 43, and 48 DAS) and the drop (in 53 DAS) were apparent in the first three generations, this pattern was not observed in the subsequent two generations (Fig. 3B). Thus, these observations suggest that the influence of advanced age on ICR rates is stronger in the first three generations, following which the influence gets weaker.

**Fig. 4:**
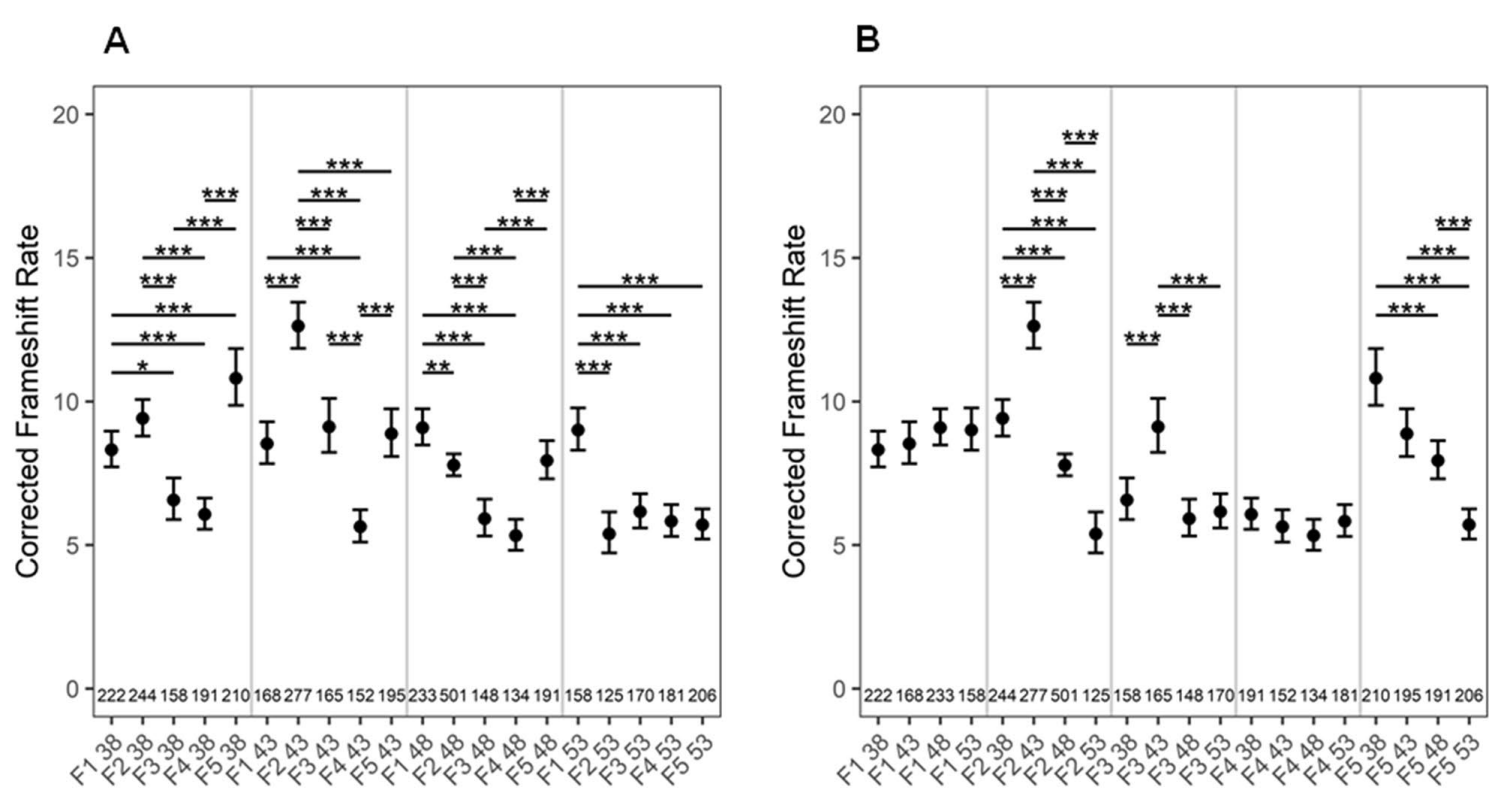
**A-B**: FS mutation rates as a function of (**A**) fixed, (**B**) increasing parental age across generations. The numbers at the bottom of the graph show the number of seedlings analyzed. *P* values were corrected for multiple testing. No asterisk denotes no significant difference. *, *P <* 0.05; **, *P* < 0.01; ***, *P* < 0.001

### Frameshift rates show a stochastic pattern as a function of constant parental age regardless of generation

To score FS mutations as a function of constant or advancing parental ages, transgenic Col-0 plants containing out-of-frame guanine repeats (G10) in the *uidA* reporter gene [20] were used. The *uidA* gene function is restored either by the addition of two or deletion of one guanine nucleotide. In the seedlings of 38, 43, or 48 DAS plants, FS rates dropped significantly from the 3rd generation and this trend persists across subsequent generations or undergoes further reduction in later generations. However, the FS rates always increased in the 5th generation (Fig. 4A). An exception to this pattern was from the seedlings of 53 DAS plants, where FS rates dropped significantly from the 2nd and lasted till the 5th generation (Fig. 4A). Like BSR/ ICR, FS rates also shoot up significantly in the 5th generation, suggesting a time component in counting the number of generations a plant has passed through self-fertilization. If FS rates were compared as a function of advancing parental age across generations, the rates remained almost constant (38/48 DAS), or gradually declined with advancing age (53 DAS) (Fig. 4B).

### Wide fluctuation in transposition rates across same or different ages/ generations

**Fig. 5:**
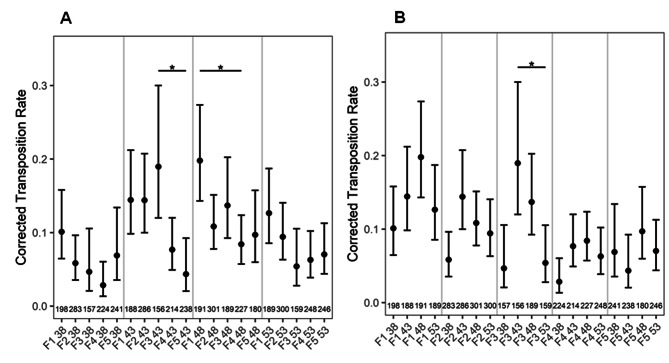
**A-B**: Transposition rates as a function of (**A**) constant (**B**) increasing parental age across different generations. The numbers at the bottom of the graph show the number of seedlings analyzed. *P* values were corrected for multiple testing. No asterisk represents no significant difference. *, *P <* 0.05

To score transposition rates as a function of constant/advancing parental age across different generations, transgenic *Arabidopsis* plants containing the transposable element *tag1* between a 35 S promoter of Cauliflower mosaic virus (CaMV) and the *uidA* gene were used [18]. *tag1* excision allows the expression of *uidA* gene, resulting in blue colored spots upon a histochemical assay. Although transposition rates as a function of constant parental age exhibited a pattern of a increase succeeded by a decrease, the differences were insignificant, with few exceptions (Fig. 5A). The transposition rates were random compared to other classes of mutations (Fig. 5A, B).

### Irrespective of age, reversion rates fluctuate widely in seedlings from fourth and fifth generations

**Fig. 6:**
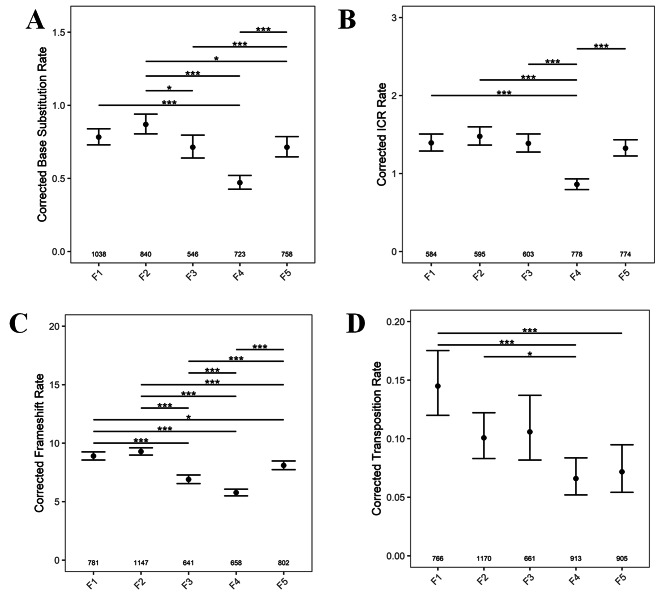
Sum total of mutation rates across generations (**A**) BSR, (**B**) ICR, (**C**) FS rates (**D**) Transposition rates in F1-F5 generation. The numbers along the X axis, show the numers of seedlings analyzed. *P* values were corrected for multiple testing. No asterisk represent no significant difference. *, *P* < 0.05; ***, *P* < 0.001

We further wanted to determine if the shift in mutation rates was significant within a certain generation, independent of age or conversely, if a significant change in reversions occurs at a certain age regardless of the generation. For this, we combined mutation rates across all ages in a particular generation (for a particular class of mutation) and independently combined the reversion rates across all five generations within a particular age for a particular class of mutation. Irrespective of parental age, BSR, ICR, and FS rates, followed similar trends with a small increase in the 2nd, a drop in the 3rd, followed by a significant reduction in the 4th and a subsequent increase in the 5th generation (Fig. 6A, B, and C). Transposition rates also decreased significantly in the 4th generation, however a similar surge in 5th generation was not observed (Fig. 6D), reiterating our previous findings where all other classes of somatic mutation rates except transpositions exhibited an increase in the 5th generation.

**Fig. 7:**
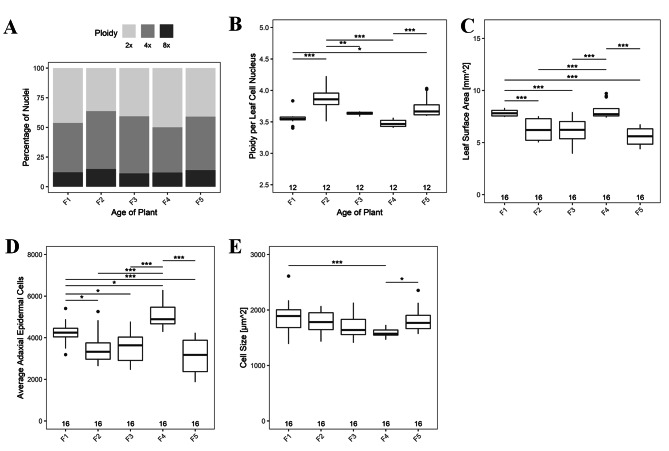
The graph representing the generation wise comparison of (**A**) total percentage of 2X, 4X and 8X nuclei, (**B**) Average ploidy per leaf per cell nucleus. (**C**) Total leaf surface area. (**D**) Average adaxial epidermal cell numbers. (**E**) Cell size of leaf. No asterisk represent no significant difference. *, *P <* 0.05; **, *P* < 0.01; ***, *P* < 0.001

Regardless of parental age, the average ploidy, the cell number, and cell size also shows a significant change in the 4th generation. Average ploidy is highest in 2nd, significantly decreased in 4th and surged in 5th generation (Fig. 7A). Ploidy per leaf cell nucleus were highest in 2nd generation (Fig. 7B). The observed leaf surface area (Fig. 7C) and adaxial epidermal cell number (Fig. 7D) were highest in the 4th and reduced drastically in the 5th generation. In contrast, there was no change in cell size in the first three generations, but dropped significantly in 4th and increased thereafter in the 5th generation (Fig. 7E).

**Fig. 8:**
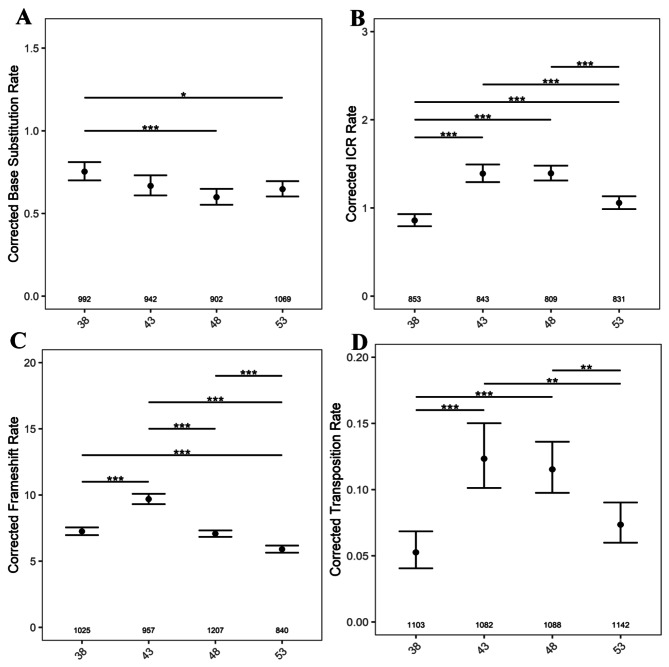
The graph representing the age-wise comparison after combining all five generations. (**A**) BSR, (**B**) ICR, (**C**) FS rates (**D**) Transposition rates in seedlings from 38, 43, 48 and 53 DAS plants. The numbers along the X axis, show the number of seedlings analyzed. *P* values were corrected for multiple testing. No asterisk represent no significant difference. *, *P* < 0.05; ***, *P* < 0.001

We then examined if mutation rates changed as a function of parental age independent of the generation by pooling mutation rates across all generations within a particular age. We observed a gradual decrease in BSR rates (Fig. 8A). Although, ICR, FS, and transposition rates, followed similar trends, with rates increasing in seedlings of 43 DAS, the subsequent decline in FS rates was apparent only from seedlings of 48 DAS. However, the significant drop in ICR, FS, and transposition rates was only in seedlings of 53 DAS plants and not from other ages (Fig. 8B, C and D).

**Fig. 9:**
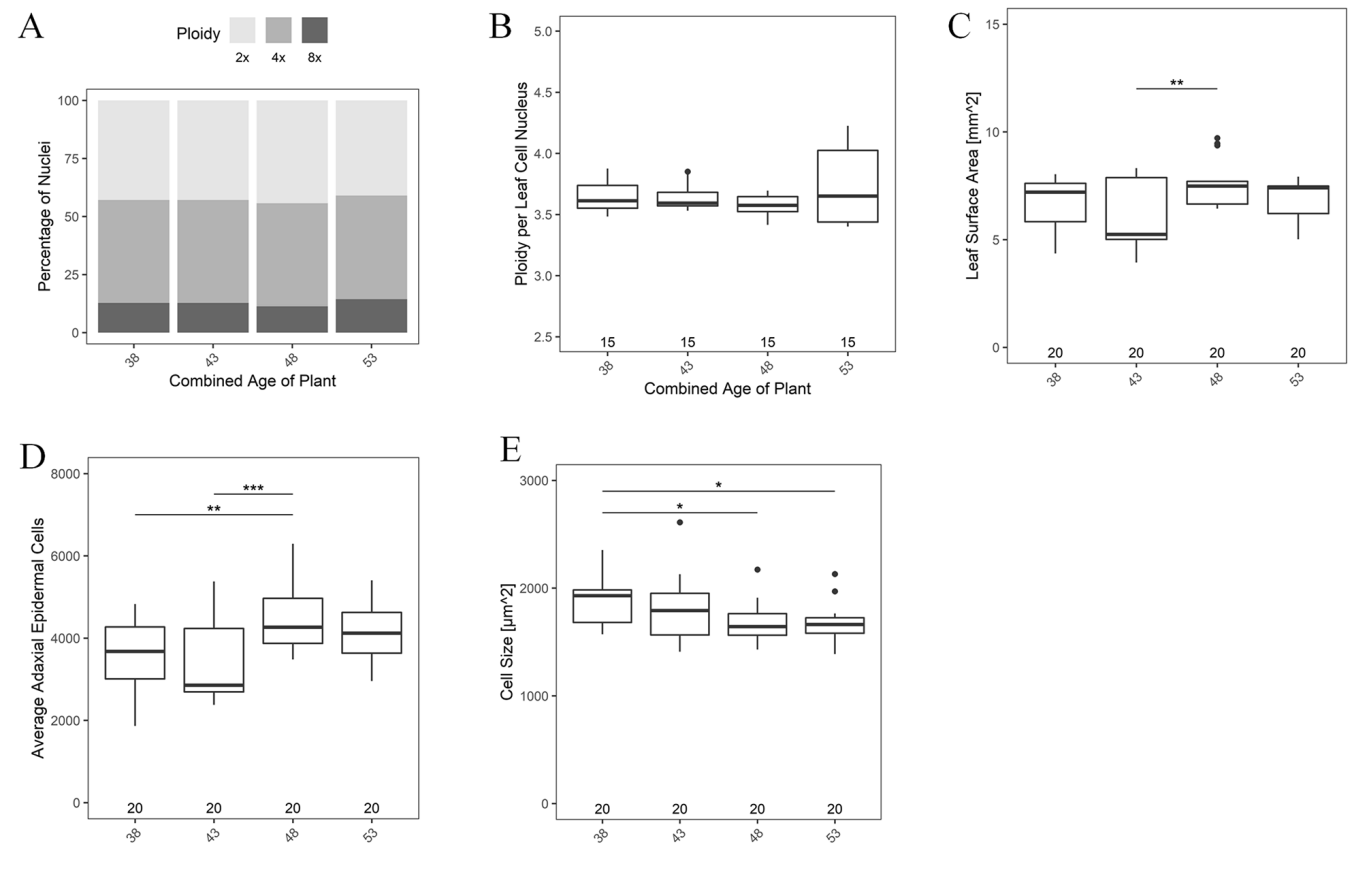
Graphs representing the age-wise comparison of ploidy, cell number, and cell size from all five generations. (**A**) Percentage of 2X, 4X, and 8X nuclei. (**B**) Average ploidy per leaf per cell nucleus. (**C**) Total leaf surface area. (**D**) Average adaxial epidermal cell number (**E**) Cell size of leaf. No asterisk represents, no significant difference. *, *P <* 0.05; **, *P* < 0.01; ***, *P* < 0.001

Regardless of the generation, no significant variation was observed in the percentage ploidy and average ploidy (Fig. 9A and B). Leaf surface area and average adaxial cell number were highest in 48 DAS (Fig. 9C and D), but cell size decreased gradually with parental age (Fig. 9E).

### Double strand DNA damage increases with increasing parental age in all generations

**Fig. 10:**
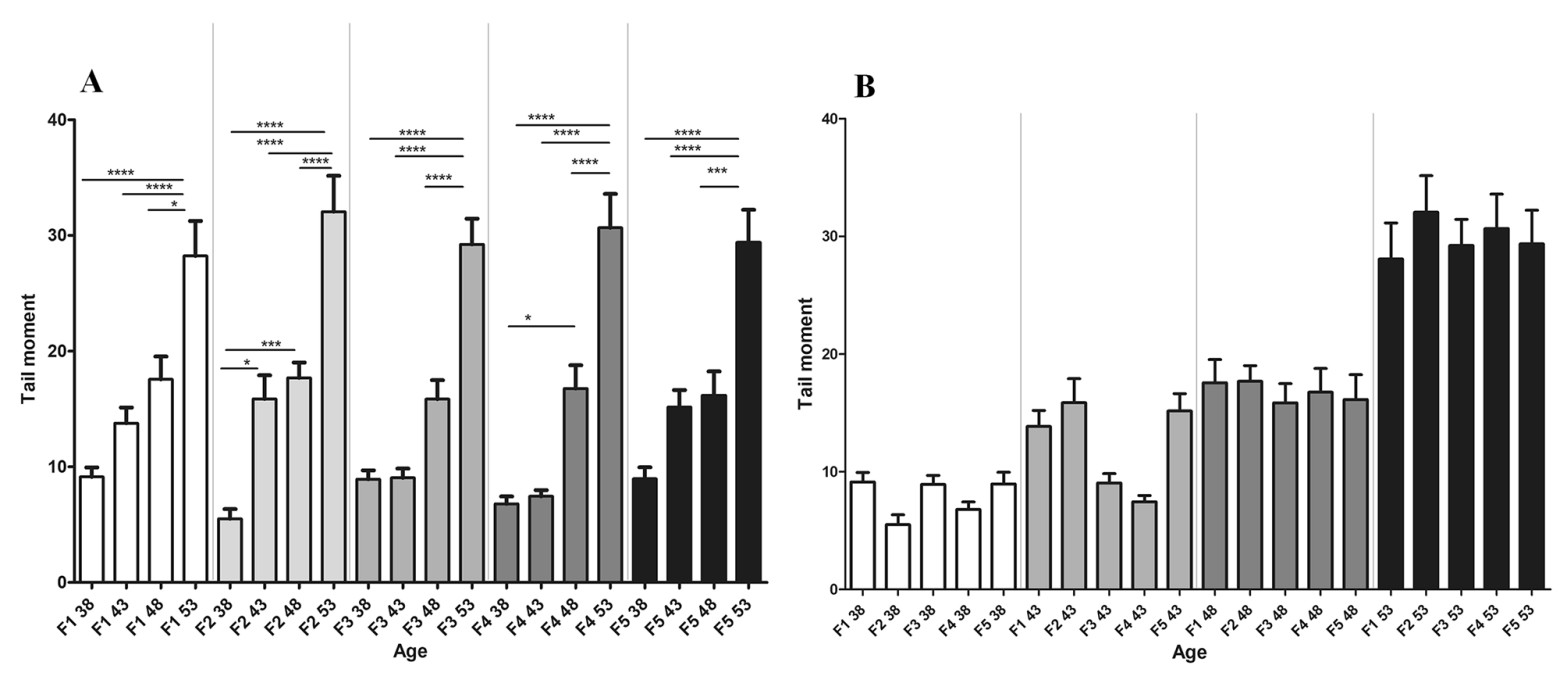
The DNA double-strand break as measured by a COMET assay. (**A**) Represents the DNA double-strand break with respect to increasing parental age in different generations. (**B**) Represents the DNA double-strand break with respect to fixed parental age in different generations. No asterisk denotes no significant differences *, *P <* 0.05; ***, *P* < 0.001; ****, *P* < 0.0001

As age is known to increase the mutation rates, we employed a COMET assay to determine if double-strand breaks (DSBs) increase in seedlings derived from parents of different ages. In this assay, damaged DNA forms a comet-like structure during migration in an electrophoresis gel [29]. DSBs were found to increase gradually in seedlings from advanced parental ages, reaching a maximum from seedlings of 53 DAS plants. The pattern of DNA double-strand breaks were similar (Fig. 10A) in the 1st, 2nd, and 5th generations and again between 3rd and 4th generations. When parental age across generations was retained, no significant change in DNA damage was observed (Fig. 10B).

### Wide variation in the expression levels of genes involved in DNA repair in the first four generations, but relatively stable expression in the 5th generation

We examined if there exists a correlation between somatic mutation rates and expression of candidate genes involved in DNA repair such as *ATM, BRCA1, RAD 51*[30, 31] in seedlings derived from fixed (Fig. S3, A, C, E, G, I, and K) and increasing parental ages across all generations (Fig. S3, B, D, F, H, J, and L). Methylation levels are also known to impact somatic mutations [32] and hence, the expression of candidate genes like *DDM1, MET1* [33, 34] were also analyzed together with *5.8 S rRNA* [35, 36] by quantitative real-time PCR.

Although few interesting patterns in gene expression across different ages/generations were observed, this did not correlate with mutation rates. For instance, in seedlings obtained from 48 DAS plants, *RAD51* expression increased in the 4th and dropped in the 5th generation, while in contrast, seedlings from 53 DAS plants exhibited low expression in the 2nd and 4th generation, but increased significantly in the 5th generation (Fig. S3, A). With increasing parental age, *RAD51* expression was significantly upregulated in seedlings from 53 DAS plants from the 3rd and 5th generation compared to F1 38 DAS plants (Fig. S3, B) but we found no correlation between the expression pattern and the observed mutation rates. Although *ATM* expression was consistently down-regulated, the drop in expression was significant in the 2nd and the 5th generation in all age groups (Fig. S3, C). However, with increasing parental age, *ATM* was down-regulated significantly in 2nd, 3^rd,^ and 5th generations, compared to F1 38 (Fig. S3, D). *BRCA1* expression was significantly down-regulated across all ages (Fig. S3, E) and with advanced parental ages, this pattern was observed only in the 4th (2.7 fold) and 5th (2.51 fold) generations compared to 38 DAS of the 1st generation (Fig. S3, F). While there was considerable heterogeneity in *MET1* expression levels across all ages/generation, the fold change (7.74 to 23.4) of *MET1* expression was striking across all ages in the 5th generation alone (Fig. S3, G). Plants of advancing ages (53 DAS) bred at the same age for five generations show an interesting *DDM1* expression pattern of an initial high, drop in 2nd, gradual increase thereafter, and another drop in the 5th generation. Except for a few, there was no change in *DDM1* expression as a function of increasing parental age (Fig. S3, J). *sRNA* shows contrasting expression patterns depending on the parental age. For instance, in 38 DAS, the expression was high initially, then dropped for the latter four generations, while in older (53 DAS) plants, the expression was initially low in the 1st generation, and underwent an increase in subsequent generations (Fig. S3, K). The wide variation in gene expression levels of different candidates analysed in the first four generations and relatively stable expression in the 5th generation may have an effect on mutation rates, which requires further investigation.

## Discussion

### Possible reasons for the differences in the mutation rates between the work of Singh et al., [17] and this study

The spontaneous reversion rates as a function of parental age is significantly different between this study and the observations of Singh et al., (22) although similar detector lines were used in both the work. Several factors could account for the observed inconsistencies between the two data sets. Previously, [17] separate plants were emasculated followed by manual self-pollination on a particular DAS. In contrast, throughout this study flowers fertilised on various DAS were used to know the parental age effect in a single plant without emasculation. Given that physical damage or mechanical stress is known to induce mutation rates [8, 37–39], emasculation may also bias the somatic mutation rates. To prevent such emasculation-induced stress responses that could potentially skew mutation rates even among the neighbouring control plants [40], we identified and tagged flowers that has just been pollinated on various DAS. Furthermore, unlike the earlier work, we plated the seeds soon after they harvested. Ideally, keeping seeds from plants of different ages/generations and staining all seedlings at the same time will remove any bias from the resulting data. However, this method would severely distort the results because seed storage is a strong determinant of mutation rates, even when stored at 4 °C (Thakur et al., data not published).

Every precaution was taken to avoid environmental perturbations that could possibly affect the reversion events. All the plants, including the controls, were grown in a highly randomised fashion in different chambers in different compartments and their physical location was changed every four days. As a result, the cumulative effect of the individual variables may explain observed variations in mutation rates.

Although we identified a comparable pattern of BSR, FS, and transposition rates as a function of parental age following manual self-pollination (Fig. S2 A, B, C, and D) and natural selfing without emasculation (Fig. S3 E, F, G, and H), the ICR rates varied significantly between this (Fig. S3) and the earlier work [17]. Earlier, the detector lines R2L1 and R3L30 were used (22) to score ICR rates and parental age had a minimal effect when R2L1 was used. However, we observed a gradual increase in ICR rates till 48 DAS when R2L1 was used. As mentioned earlier, several factors could account for the observed variations.

The reversion rates were found to be highly different, even among control plants from the same lab, but in different experiments [41–43]. For example, in line 11 reporting homologous recombination, the reversion rates from three different studies in the controls grown in identical environmental conditions were found to be 1.9, 2.7, and 2.5, whereas in line 651, the reversion rates were 0.21, 0.74, and 0.7 in the controls. In all three studies, homozygous detector lines were used and seedlings used were of the same age sets also. Similarly, using ICR detector lines IC1 and IC9, independent observations from the same lab reported distinct reversion rates for each line [8, 44]. Thus, for a given line the frequency of reversion events was highly inconsistent even among the controls and a number of parameters such as seed storage age, parental age, and the number of generations the plants harbouring GUS had gone through selfing at a given DAS could have biased the reversion events. Given these considerations, seed storage was avoided and seeds were plated soon after harvesting.

The seeds were obtained from various labs and the number of times the plants were selfed following transformation is unknown. The persistence of stress memory effect in plants is known to diminish away after four generations [8]. Furthermore, the GUS assay replicates were grown at separate times, but within the same chambers, minimising the influence of environmental conditions in influencing mutation rates.

By whole genome sequencing of *A. thaliana* produced from a single seed descent, somatic mutation rates compared in two widely separated generations [45] and it was found that spontaneous mutations are biased towards G:C than A:T and the occurrence of such mutations are high near transposable elements and pericentromeric/centromeric regions [7]. However, it is unknown whether the parental age was retained in the successive generations in that study [45]. Although sequencing approaches are insightful, it has serious limitations in identifying this particular class of rare somatic mutations. Because spontaneous reversion events are rare, sequencing techniques will not help to identify them. Our findings strongly show that somatic mutation rates follow a unique pattern that is significantly influenced by the number of generations a plant has gone through selfing at a given age. Despite a limited number of generations studied, the evidence strongly suggests that somatic mutation rates follow a pattern that is substantially impacted by parental age and generation time. This study shows that parental age alone contributes to fluctuations in mutation rates, with different ages showing different patterns of fluctuation. Such stochastic processes are a part of successful evolutionary adaptations in unpredictably changing environments [46]. The expression pattern of DNA repair genes such as *RAD51, ATM, BRCA1*, and *sRNA* were random in the first four generations, but became rather stable in the fifth generation (Fig. S4). The number of generations can be counted by fluctuations followed by stable gene expression that also impacts the mutation rates, since there is a surge in C to T transition rates every fifth generation.

Our observations on mutation rates dropping gradually in every generation until they reach their lowest point in the fourth after which it goes up in the fifth generation is similar to the inference of ‘Greer and colleagues’ on transgenerational epigenetic inheritance of longevity in *C. elegans* [47] where low H3K4me3 (histone H3 lysine 4 trimethylation) expression levels was found to be associated with longevity in the first three consecutive generations and higher expression levels was correlated with poor longevity in the latter two generations [47–49]. Similarly, it has been reported that in *A. thaliana*, reduced mutation rates are attributed by H3K4me3 expression [50].

Another proposed mechanism for transgenerational lifespan inheritance could be the transmission of small interfering RNAs (siRNA). Indeed, heritable gene silencing has been seen in *C. elegans* in response to double-stranded RNA treatment, with silencing phenotypic effects lasting up to F3 or F4 generations [49]. Furthermore, a study in *C. elegans* found that small-interfering RNAs produced from a viral genome after infection can be passed to uninfected worm progeny in a transgenerational manner, [51] potentially giving adaptive benefits to the organisms. Thus, H3K4me3 and siRNA expression can play a significant role in transgenerational inheritance indicating the probable role of H3K4me3 and siRNA in age dependent information which persists for few generations.

If such a transgenerational epigenetic inheritance process also occurs in *Arabidopsis* that will most likely affect gene expression and mutation rates according to the parental age. Cytosine methylation is associated with increased C→T base substitution events, and methylcytosine is prone to deamination [52]. A high degree of DNA methylation may result in poor gene expression [53], and genes that transcribe at low levels are likely to have low mutation rates as well [54]. DNA methylation levels are known to be higher in older plant tissues than in younger plant tissues [55–57], although other reports contradict the results [58–60]. Cytosine DNA methylation is critical for genome integrity and gene expression regulation [61] and is also known to increase neighbourhood mutations in *Arabidopsis* somatic cells [62]. This indicates that methylation occurs in a context-dependent manner during development [63, 64]. Our findings reveal that seedlings from younger and older parents had higher C→T transition rates than seedlings from middle-aged parents.

The expression of *DDM1* (Fig. S3, J) can reciprocally be correlated with reversion rates in seedlings from middle-aged parents (43 and 48 DAS) in the first and third generations, i.e. lower the expression, higher is the mutation rates and a similar trend in the fourth and fifth generations, supporting the notion that *DDMI* plays a significant role in higher C→T transition from younger and older parents (Fig. 2B).

Our findings suggest that seedlings of 38, 43, and 48 DAS plants had higher ICR events than seedlings of 53 DAS plants (Fig. 3B). Despite the fact that DSBs increase with parental age, ICR rates were low in seedlings obtained from 53 DAS plants, which could be attributed to increased non-homologous end-joining (NHEJ) with plant age [50]. The expression of the Ku70 protein, known to be involved in NHEJ, was found to increase with plant age [5]. Although ICR rates drop in seedlings derived from aged parents, NHEJ levels increase. This effect that may be passed on in a particular parental age in the latter generations justifying the observed increase in DSBs in the descendants of older parents.

Plants with a prolonged life span are known to have low cell division rates and as a result, low mutation rates [65]. It is also likely that cell division rates in the reproductive meristem is not consistent throughout the ages. Furthermore, DNA replication is known to be independent of life span, implying that older plants may not pass on more mutations to their offspring than younger plants [65]. It is well understood that the accumulation of somatic mutations with age results in the inactivation of random genes crucial for somatic cell function [29, 66, 67]. As a result, epigenetic inheritance, which could be mediated by DNA methylation or small RNAs, could be a key mechanism driving the observed transgenerational effects [68–71].

## Materials and methods

### Plant growth conditions

Prior to planting, *Arabidopsis thaliana* (Columbia) seeds were surface sterilized with 70% (v/v) ethanol followed by 0.5% (v/v) bleach treatment for 3 min. The seeds were washed thrice with sterile water and plated on autoclaved MS medium (with 3% [w/v] Suc), pH 5.7, and containing 0.05% (v/v) Plant Preservative Mixture (Biogenuix Medsystem Pvt. Ltd.) and kept at 4 °C for 48 h in the dark for synchronized seed germination. The plates were transferred to plant growth chambers (Percival, USA) that had a uniform light intensity of 8,000 lx (under a 16-h-light/8-h-dark cycle). The temperature of the growth chamber (Percival CU-36L6) was maintained at 22 °C throughout, and the humidity was set to 80%. Three-week-old seedlings were transferred from MS plates to soil and grown again in a growth chamber (Percival AR-36L3). The soil consisted of equal proportions of garden soil, peat, perlite, and vermiculite. Plants were grown in a randomized manner in the growth chambers and at frequent intervals, their positions were changed.

### Somatic mutation detector lines

All the detector lines used in this work are in Col-0 background. The base substitution detector line 1,390T→C was a gift from Anna Depicker, University Ghent University, Ghent, Belgium [21]. Transgenic ICR lines (R2L1) carrying inverted catalase introns (418 bp) in the *uidA* gene and the frameshift detector line (G10) were a gift from Francois Belzile, Universite´ Laval, Quebec, Canada [24, 26]. The detector line harboring the *Tag1* element was a gift from Nigel M. Crawford, University of California, San Diego, USA [18].

### Identification of self pollinated flowers from plants of different DAS

Flowers self-pollinated on different DAS (38, 43, 48, and 53) were identified from the same plant and marked with different colored threads after anthesis. The plants representing different DAS were maintained as independent lineages in the succeeding generations. In total, twenty-four plants of a particular age were represented in each generation. The transgenic detector lines were generated by different groups [18–21] and have been tested on several occasions [15, 72, 73]. The somatic mutation detection using such lines [74] rely on the reversion of a mutated or truncated *uidA* gene all driven by a CaMV-35 S promoter and only a specific reversion event alone would lead to functional GUS formation. Upon histochemical staining of the seedlings, the reversions could be visualized as blue coloured spots. For example, in line M4T4 (1390T→C), the gene *uidA* was modified such that thymine (T) was replaced with cytosine (C) at a specific site so that the expression is disrupted. A spontaneous C to T transition (BSR) at the mutated site would restore the ORF of *uidA* and thus a functional GUS would be formed. Similarly, in the frameshift detector line G10 [20], a repeat of ten guanine (G) nucleotides was engineered within the *uidA* gene. Either upon the excision of one ‘G’ or by the addition of two ‘G’s, the frame could be restored. In the ICR detector line R2L1 [19], a truncated *uidA* with partially overlapping catalase introns was constructed over two arms of the sister chromatid and a recombination event between similar sequences of the catalase intron would result in a functional *uidA* gene, leading to the GUS expression [19]. Lastly, to detect transposition events, a *tag1* element engineered between a global CaMV35S promoter and the *uidA* sequence was used and the precise transposon excision would result in functional transcription of *uidA* and thus GUS formation [18]. Matured seeds were harvested and germinated on MS plates.

### Histochemical staining for GUS activity

The GUS staining was carried out on intact 3-week-old seedlings as described by Jefferson et al., in three biological replicates [75] that had previously been grown in MS agar plates with the antibiotic. Plant sections were not used for the analysis. The entire lot of seedlings were immersed in freshly prepared 5-Bromo-4-chloro-3-indolyl-D-galactoside (X-Gluc, BIOSYNTH, B-7300) solution (pH of 7). Following a 15-minute desiccation period at room temperature, the samples were incubated at 21^0^ C in dark conditions for 48 h. Subsequently, the GUS staining solution was removed and the seedlings were rinsed and cleared with 70% ethanol. The GUS revertant spots were seen primarily in the leaves. The error rates were reduced by staining all the seedlings of a particular age at the same time in similar conditions.

The GUS staining solution contains:

**1)** 100 mM Sodium phosphate (NaPO_4_, pH 7.0), **2)** 0.2% Triton X-100, **3)** 10 mM EDTA **4)** 100 mM 5-bromo-4-chloro-3-indolyl glucuronide (X-Gluc, Biosynth Switzerland) **5)** 0.5 mM potassium ferricyanide K_3_Fe(CN)_6_ and 0.5 mM potassium ferrocyanide K_4_Fe(CN)_6_. The pH of the final solution was maintained at 7. X-Gluc stock (1mM) solution was prepared by dissolving 5 mg of X-gluc in 1 ml of *N*,*N*-dimethylformamide.

The total number of seedlings analyzed is indicated in the Figures. The blue colored GUS reversions were counted using a stereozoom microscope (Leica KL300, Germany).

### Estimating correction factors to calculate mutation rates

The mutation rates were not corrected for replication cycle, but for the genome number. Mutation rates were calculated by dividing the average number of GUS spots per plant by the copy number of the transgene [22]. Since the total number of cells and the average ploidy per nucleus differ between seedlings from different generations, the total genome number will not be the same also. Hence, mutation rates were corrected by accounting for changes in the number of adaxial epidermal cells of the fourth true leaf and the average ploidy in 3-week-old seedlings (derived from F2, F3, F4 and F5 generations of 43, 48, and 53 DAS). This was compared with the adaxial epidermal cells of the fourth true leaf and ploidy levels in seedlings derived from F1 38 DAS plants. The correction factor was calculated using the formula [17].

$$TITER{\rm{ }} = {\rm{ }}\left( {{P_H} \times {C_H}} \right)/{\rm{ }}{P_Y} \times {C_Y}$$

To compare differences in mutation rates as a function of plant age, seedlings derived from young parents (F1 38 DAS) were considered [17]. *P*_H_ is the average ploidy per nucleus in 3-week-old seedlings from 43, 48, and 53 DAS plants of F2, F3, F4, and F5 generation. *C*_H_ is the average number of adaxial epidermal cells in the fourth true leaf of seedlings derived from 43, 48, and 53 DAS plants of F2, F3, F4, and F5 generation. *P*_Y_ is the average ploidy per nucleus in 3-week-old seedlings from F1 38 DAS. *C*_Y_ is the average number of adaxial epidermal cells in the fourth true leaf of seedlings derived from F1 38 DAS plants.

Corrected mutation rate = GUS/titer.

Where GUS is the average number of blue spots per plant.

### Statistical analysis

A total of 3,905; 3,336; 4,027; and 4,415 seedlings were examined to detect BSR, ICR, FS, and transpositions, respectively. The GUS reversions were random and do not follow a normal distribution and hence ANOVA test was avoided. The number of GUS spots are count data and to account for overdispersion in the data, we chose a Quasi-Poisson generalized linear model (GLM) with the log link function [76]. The log of the correction factor for cell number and ploidy per leaf cell nucleus was integrated into the models as a fixed intercept. In all GLMs, the data from the groups were used for multiple comparisons. Correction for multiple testing was done to keep the family-wise error rate at 5% [77]. *P* values were adjusted with a single-step method that considers the joint multivariate *t* distribution of the individual test statistic [78]. The results are presented with a two-sided *P* value adjusted for multiple comparisons. All the statistical analysis was done using R [79]. For multiple comparisons, *P* values were modified by multcomp package R [78]. Graphs were produced with ggplot2 [80].

### Cell size and cell number analysis by scanning electron microscopy

For SEM, a wax impression of plant tissue was prepared according to the protocol of [81]. The fourth true leaf of a 3-week old *Arabidopsis* seedling derived from parents of different ages was dissected, and as suggested by the manufacturer, the two components of waxy dental material were deposited on the leaf to generate an impression (Coltene PRESIDENT light body, Coltene AG, Altstatten, Switzerland). After 5 min, when the wax had hardened, the leaves were gently removed. This leaf mould was used for sample preparation with Spurr resin, and the resin with the leaf impression was taken out carefully from the mould, and the sample was coated with gold using Polaron Range sputter coater (Quorum Technologies). The coated resins were mounted onto a SEM stub with a double-sided carbon tape to capture the images (FEI Quanta 400-F SEM (FEI Company, USA) under 20 kV voltage and 70 Pa pressure (Fig. S1 A,B,C, and D). The adaxial leaf surface area was calculated by using the SEM images captured at 50X magnification [17]. Independently, SEM images were taken at different positions of the leaf at 500X magnification. The average cell size was calculated by dividing the number of cells observed in an area of 500X magnification at different positions of the leaf. To calculate the total number of adaxial epidermal cells, the total area of the leaf was divided by a fixed area of 500X magnification and multiplied by the number of cells present in an area of 500X magnification. ImageJ (NIH, USA) software was used for counting the total number of cells and the total area of the leaf. Four biological replicates were considered to determine the average number of adaxial epidermal cells of the 4th true leaf.

### Ploidy analysis by flow cytometry

Ploidy analysis was performed as per the protocol of Doležel et al., [82]. The fraction of nuclei with 2X, 4X, and 8X ploidy was estimated together with the average number of genomes per nucleus from seedlings of different parental ages. Approximately 60 mg of leaf tissue of 3-week-old seedlings was finely chopped with a razor blade in a glass Petri dish containing 1 mL of ice-cold nuclei extraction buffer (Sysmex CyStain^™^ PI Absolute P KIT). The chopped tissues were filtered with a 20 μm nylon filter (CellTrics® filters, Sysmex). Three biological replicates were carried out for flow cytometry. To stain the nuclei, 2 ml of staining buffer (provided with the kit) containing propidium iodide and RNase in 1:2 ratio was mixed to the filtrate and kept for an hour at room temperature. A sample derived from Tomato (*Solanum lycopersicum* ‘Stupicke’) was used as a control. After the incubation period, the samples were analyzed using a Partec CyFlow® Ploidy analyser (Sysmex). Data analysis was carried out with CyView Software (Sysmex).

### RNA isolation and quantitative real-time PCR

Using the Trizol reagent (Invitrogen, USA), total RNA was isolated from 21-day old seedlings of wild type Columbia-0 (Col-0) obtained from plants of different ages in five consecutive generations. cDNA was synthesized using 2 µg RNA, random hexamers, and High Capacity cDNA Reverse Transcription Kit (Applied Biosystems, USA). Quantitative real-time PCR was done in triplicate using the DyNAmoTM Flash SYBR Green qPCR kit (QuantStudio (TM) 7 Flex System). The primers for amplifying *ATM, BRCA1, RAD51, DDM1, MET1*, and *sRNA* (5.8 S *rRNA* and 25 S *rRNA* with 18 S rRNA fragment) are listed in Table S1. The expression levels of glyceraldehyde-3-phosphate dehydrogenase (*GAPDH*) mRNA was used as an internal standard. The relative gene expression in each age and generation was determined by calculating 2^(−ΔΔCt)^ as described previously [83].

### Neutral COMET assay

To measure ds DNA breaks, a COMET assay was performed using the Oxiselect Comet Assay Kit from Cell Biolabs, Inc. USA. 3 week old Col-0 seedlings of 38, 43, 48, and 53 DAS from all five generations were chopped using a razor blade in 1 mL of Otto I solution. Subsequently, the sample was filtered with a 20 μm nylon filter (CellTrics® filters, Sysmex), and the filtrate was centrifuged at 300 rpm for 5 min. The pellet was dissolved in phosphate-buffered saline (PBS) containing 20 mM EDTA. The dissolved sample was mixed with warm low-melting agarose in a ratio of 2:5 and poured onto a slide coated with normal agarose. The sample was covered with a glass coverslip, stored at 4 °C for 15 min and after removing the coverslip, the slides were transferred to a chamber filled with chilled lysis buffer for 30 to 60 min at 4 °C. The slides were then transferred to another chamber filled with cold alkaline solution (NaOH and EDTA, pH 10) for 30 min at 4 °C in the dark. The slides with the sample were treated with chilled Tris-borate/EDTA buffer for 5 min and transferred to a horizontal electrophoresis chamber containing the same buffer. Electrophoresis was carried out at 1 V cm^− 1^ for 15 min. Thereafter, the slides were gently rinsed thrice with deionized water, followed by rinsing with 70% (v/v) ethanol for 5 min. After air drying, the sample was stained with vista green and kept for 15 min in dark at room temperature. Then, the COMETS were observed using an upright fluorescence microscope (Nikon Eclipse 80i) fitted with a fluorescein isothiocyanate (FITC) filter. We analysed around 50–55 Comet using a free online tools available from CaspLab. One way ANOVA with a 95% confidence interval of difference was used for the statistical analysis carried out by prism (Version 5) software (USA).

## Electronic supplementary material

Below is the link to the electronic supplementary material.

Supplementary Material 1

Supplementary Material 2

Supplementary Material 3

Supplementary Material 4

Supplementary Material 5

Supplementary Material 6

Supplementary Material 7

## Data Availability

The datasets analysed are available from the corresponding author.
