# Supplementary material for: Persistence of parental age effect on somatic mutation rates across generations in Arabidopsis
Source: BMC Plant Biol. 2023 Mar 22;23:152. doi: 10.1186/s12870-023-04150-w (PMC10031922; doi:10.1186/s12870-023-04150-w)
Supplement: Supplementary file 1 — Supplementary Material 1 [file 12870_2023_4150_MOESM1_ESM.docx]

| **Epidermal cells** | | | | | **Ploidy per nucleus** | | | | |
| --- | --- | --- | --- | --- | --- | --- | --- | --- | --- |
| **Age** | **n** | **Median** | **CIQR** | **Relative cell number** | **n** | **Median** | **CIQR** | **Relative ploidy** | **Correction factor** |
| F1 38 | 4 | 4134.841645 | 0.074743099 | 1 | 3 | 3.557 | 0.0402 | 1 | 1 |
| F1 43 | 4 | 4092.023519 | 0.111139791 | 0.989644555 | 3 | 3.577 | 0.0056 | 1.005622716 | 0.995209 |
| F1 48 | 4 | 4175.036919 | 0.119608416 | 1.009721116 | 3 | 3.544 | 0.0051 | 0.996345235 | 1.006031 |
| F1 53 | 4 | 4673.600815 | 0.120248471 | 1.130297413 | 3 | 3.425 | 0.0158 | 0.962890076 | 1.088352 |
| F2 38 | 4 | 3686.811979 | 0.025377695 | 0.891645266 | 3 | 3.866 | 0.0013 | 1.086870959 | 0.969103 |
| F2 43 | 4 | 2791.664624 | 0.097476684 | 0.675156358 | 3 | 3.841 | 0.0021 | 1.079842564 | 0.729063 |
| F2 48 | 4 | 4565.716472 | 0.165895059 | 1.104205884 | 3 | 3.558 | 0.0121 | 1.000281136 | 1.104516 |
| F2 53 | 4 | 3091.480092 | 0.081892202 | 0.747665898 | 3 | 4.209 | 0.0026 | 1.183300534 | 0.884713 |
| F3 38 | 4 | 3310.743566 | 0.177374671 | 0.800694162 | 3 | 3.639 | 0.0036 | 1.023053135 | 0.819153 |
| F3 43 | 4 | 2628.679586 | 0.078037259 | 0.635738877 | 3 | 3.594 | 0.0053 | 1.010402024 | 0.642352 |
| F3 48 | 4 | 4049.544556 | 0.109844988 | 0.979371135 | 3 | 3.654 | 0.0027 | 1.027270171 | 1.006079 |
| F3 53 | 4 | 4223.011129 | 0.160442252 | 1.021323545 | 3 | 3.651 | 0.0044 | 1.026426764 | 1.048314 |
| F4 38 | 4 | 4650.910075 | 0.02811808 | 1.12480972 | 3 | 3.486 | 0.0055 | 0.980039359 | 1.102358 |
| F4 43 | 4 | 5036.341781 | 0.046995477 | 1.218025311 | 3 | 3.556 | 0.0048 | 0.999718864 | 1.217683 |
| F4 48 | 4 | 6083.262136 | 0.04447478 | 1.471220099 | 3 | 3.42 | 0.0035 | 0.961484397 | 1.414555 |
| F4 53 | 4 | 4677.228272 | 0.066146604 | 1.131174703 | 3 | 3.43 | 0.0061 | 0.964295755 | 1.090787 |
| F5 38 | 4 | 2214.953776 | 0.072334608 | 0.535680436 | 3 | 3.605 | 0.0025 | 1.013494518 | 0.542909 |
| F5 43 | 4 | 2718.777308 | 0.057053435 | 0.657528762 | 3 | 3.668 | 0.0044 | 1.031206073 | 0.678048 |
| F5 48 | 4 | 3930.042162 | 0.049012705 | 0.950469812 | 3 | 3.648 | 0.0132 | 1.025583357 | 0.974786 |
| F5 53 | 4 | 3813.991401 | 0.053696917 | 0.922403257 | 3 | 4.016 | 0.0042 | 1.129041327 | 1.041431 |

Table S1. The mutation rates were normalized by factoring the differences in cell number of the 4th true leaf and ploidy per nucleus from plants of different ages/generations. The correction factor was derived from a five-generation study of adaxial epidermal cell count and ploidy per nucleus of leaf cells. The Interquartile Range Coefficient (CIQR) was calculated as a non-parametric variance measurement in the style of the variance coefficient. The relative number of cells and the relative ploidy are the normalization values for generations F2, F3, F4, and F5 compared to F1, and for older ages (43, 48, and 53 DAS), the comparison was with 38 DAS plants. The correction factor was determined by combining the two values for standardization and was introduced before correcting the number of GUS spots. ‘n’ is the number of plants analyzed and median is the median of measurements.
