# Supplementary material for: Persistence of parental age effect on somatic mutation rates across generations in Arabidopsis
Source: BMC Plant Biol. 2023 Mar 22;23:152. doi: 10.1186/s12870-023-04150-w (PMC10031922; doi:10.1186/s12870-023-04150-w)
Supplement: Supplementary file 2 — Supplementary Material 2 [file 12870_2023_4150_MOESM2_ESM.docx]

| **S. no.** | **Gene** | **Primer sequence** |
| --- | --- | --- |
| **1** | ***RAD51*** | FP 5’TCCATAGCTCAGTGATAGAGC3’ |
|  |  | RP 5’GATTAGTGAACTACTTGACGC3’ |
| **2** | ***MET1*** | FP 5’TGACGAAAAAACGGACCCGATA3’ |
|  |  | RP 5’TGGTATACTACAGGTGAAACTC3’ |
| **3** | ***DDM1*** | FP 5’AAGGGACGACGGAGACGACTGTT3’ |
|  |  | RP 5’CACCAAGAAGAACAGCGAGCGAT3’ |
| **4** | ***BRCA1*** | FP 5’CGCAGCACTTGGTTGTTACA3’ |
|  |  | RP 5’GCTGAGAGTGCAGTGGTTCT3’ |
| **5** | ***ATM*** | FP 5’GAAGTGGTGGCCAGGAGAAG3’ |
|  |  | RP 5’TCGAAGAAGCGAACCACGAA3’ |
| **6** | ***GAPDH*** | FP 5’GGTCACCATGGCTTCGGTTA3’ |
|  |  | RP 5’AGTCTGGAAGGAGACGATGGA3’ |
| **7** | **5.8S rRNA** | FP 5’GCAACGCCTCGACCTATTCT3’ |
|  |  | RP 5’TTCTAGGTTAGCGCGCAGTT3’ |

Table S2. List of primers used for real-time PCR and their sequence.
