# Supplementary figures and images for: Persistence of parental age effect on somatic mutation rates across generations in Arabidopsis
Source: BMC Plant Biol. 2023 Mar 22;23:152. doi: 10.1186/s12870-023-04150-w (PMC10031922; doi:10.1186/s12870-023-04150-w)

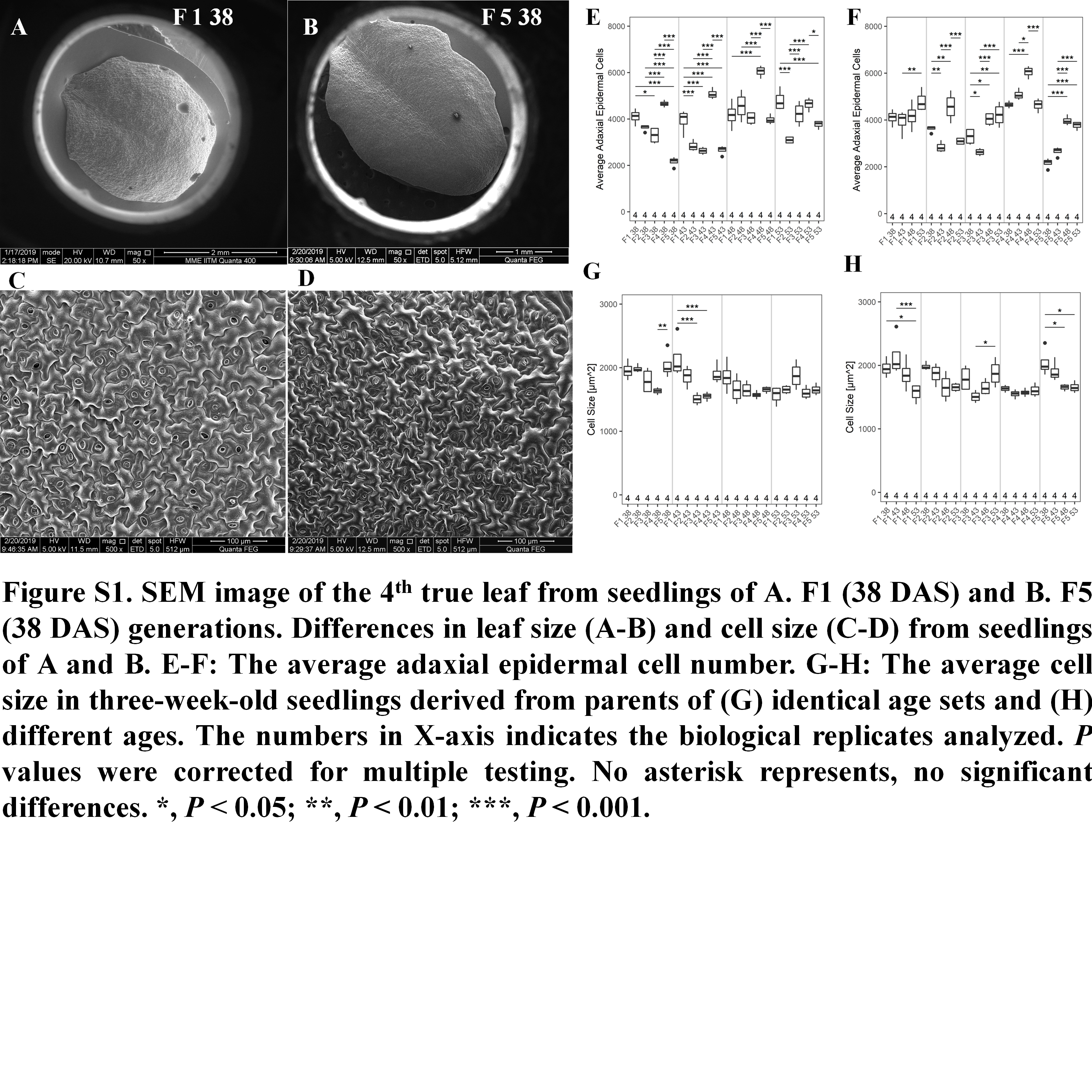

Supplement: Supplementary file 3 — Supplementary Material 3 [file 12870_2023_4150_MOESM3_ESM.tif]

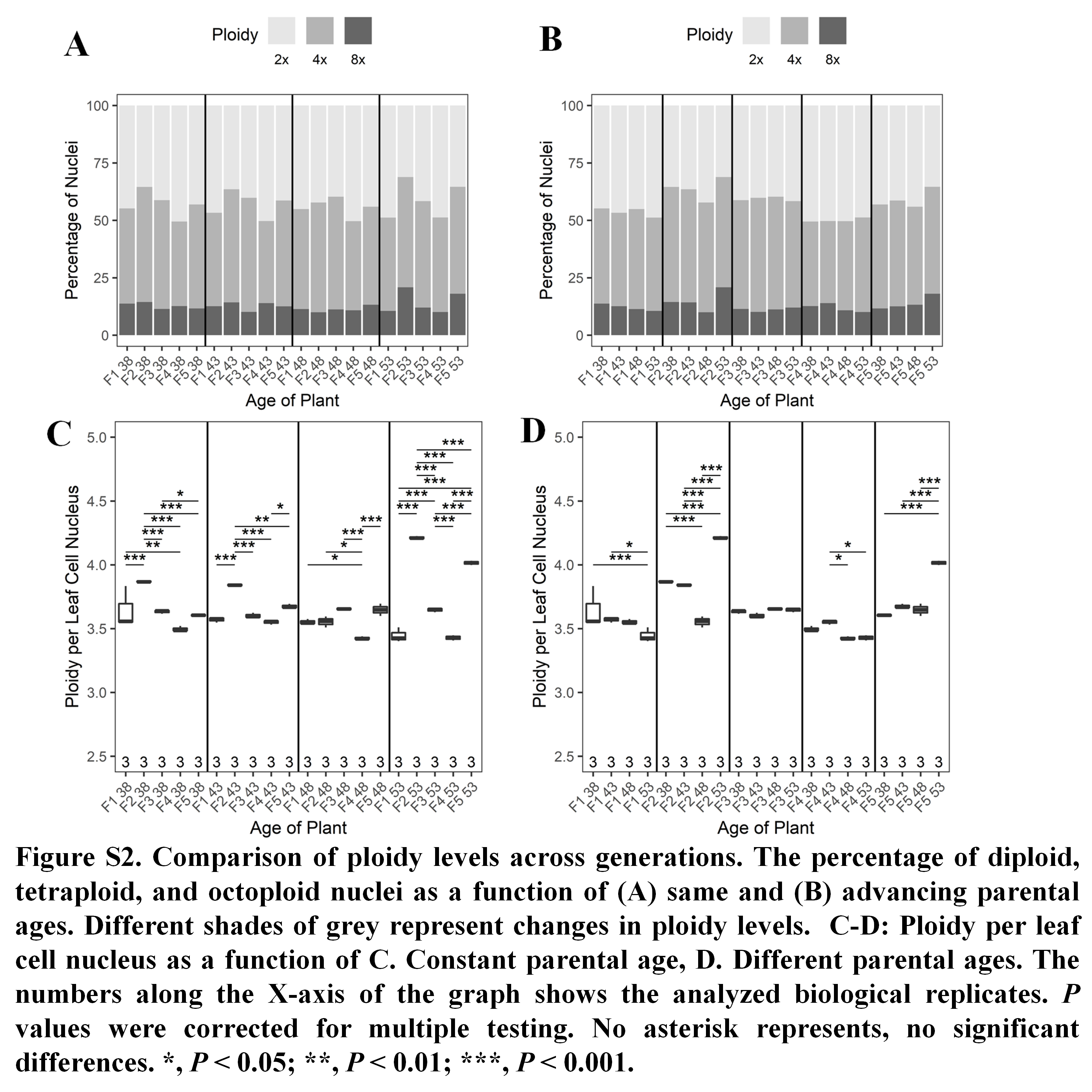

Supplement: Supplementary file 4 — Supplementary Material 4 [file 12870_2023_4150_MOESM4_ESM.tif]

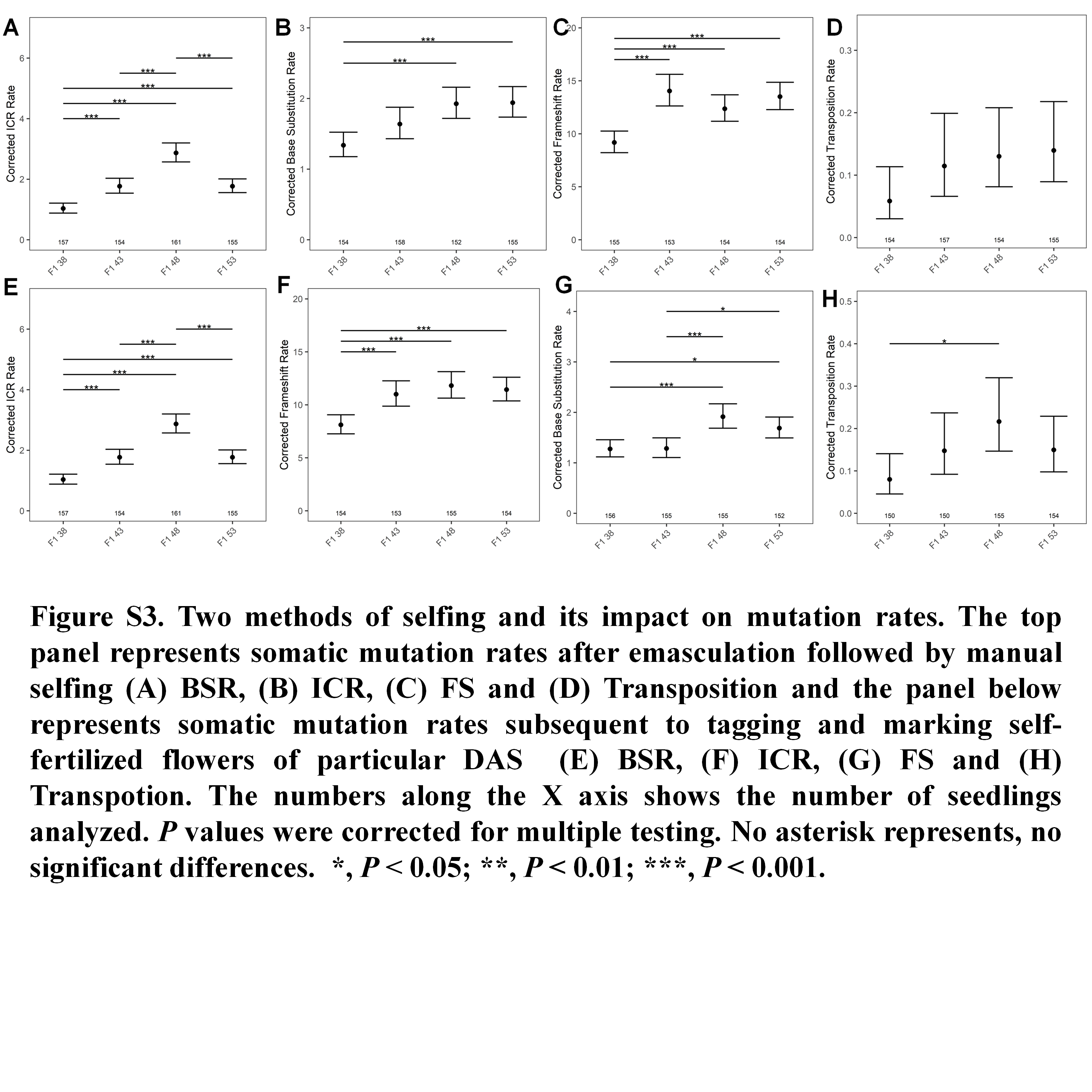

Supplement: Supplementary file 5 — Supplementary Material 5 [file 12870_2023_4150_MOESM5_ESM.tif]

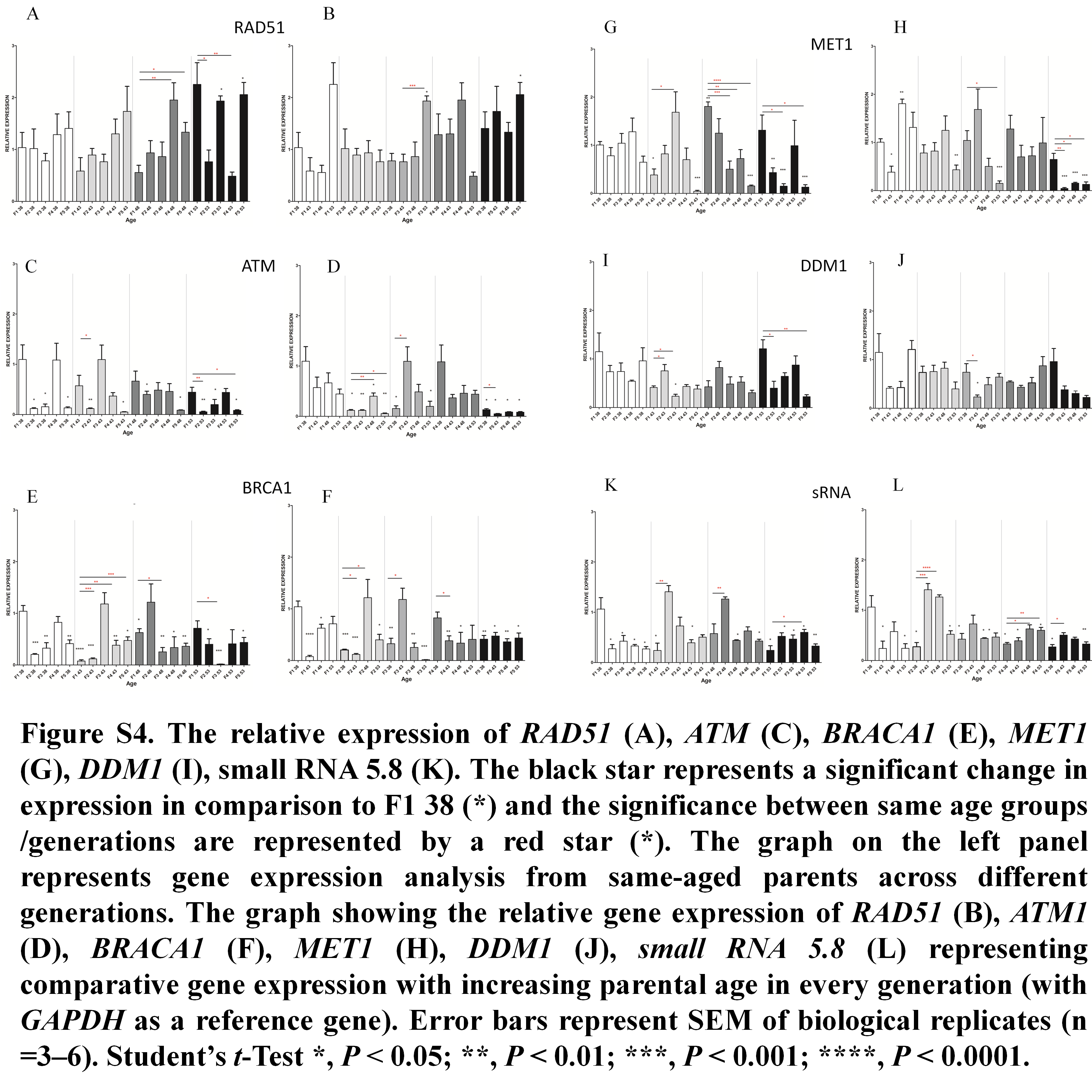

Supplement: Supplementary file 6 — Supplementary Material 6 [file 12870_2023_4150_MOESM6_ESM.tif]

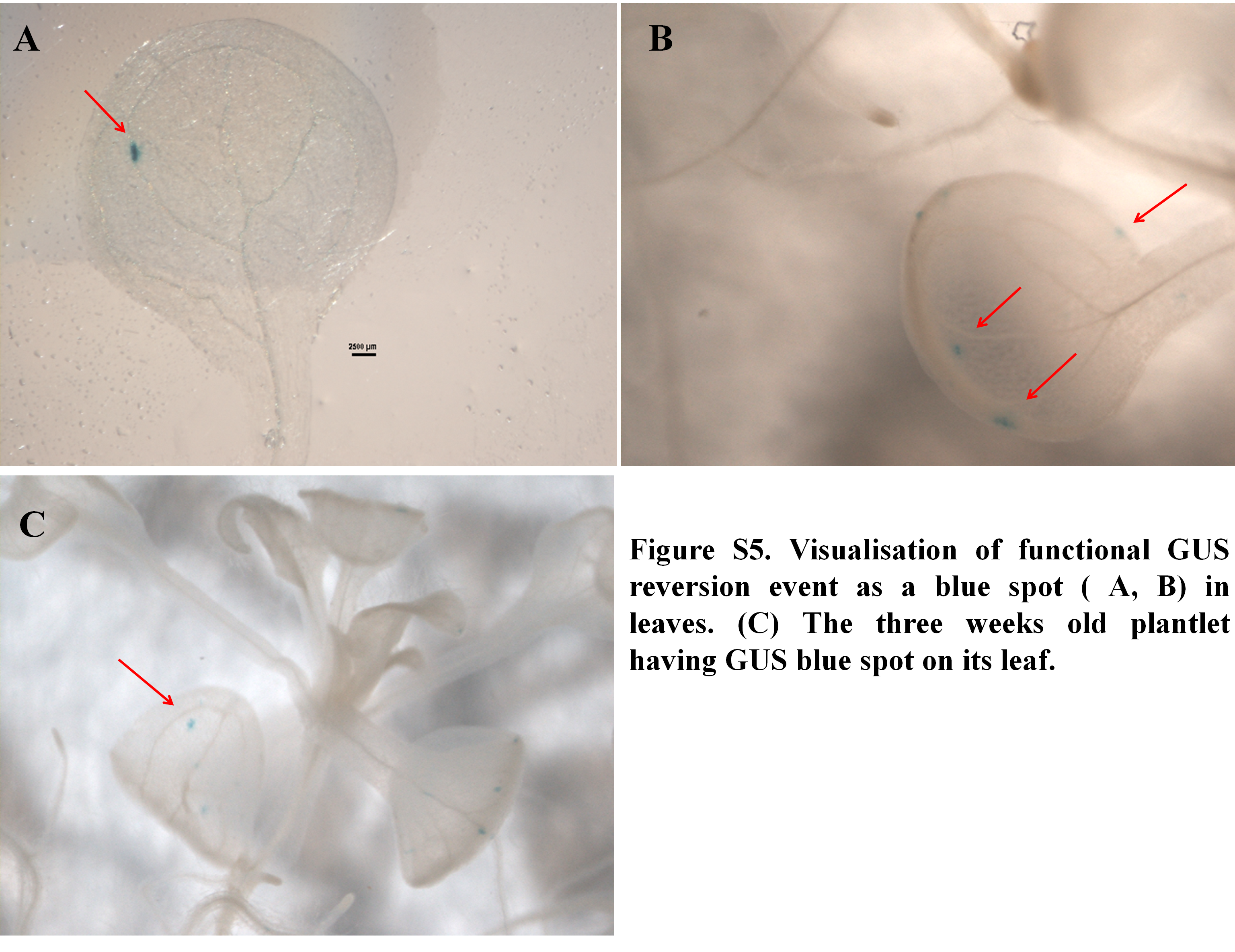

Supplement: Supplementary file 7 — Supplementary Material 7 [file 12870_2023_4150_MOESM7_ESM.tif]
